# Coexistence of Plasma Cell Dyscrasia with Prefibrotic Stage of Primary Myelofibrosis: A Case Report

**DOI:** 10.5402/2011/404057

**Published:** 2011-05-19

**Authors:** George Tsirakis, Peggy Kanellou, Maria Kaparou, Andrew Passam, Amalia Zampoulaki, Kostas Stylianou, Michael G. Alexandrakis

**Affiliations:** ^1^Department of Haematology, University Hospital of Heraklion, 71110 Heraklion Crete, Greece; ^2^Department of Nephrology, University Hospital of Heraklion, 71110 Heraklion Crete, Greece

## Abstract

*Introduction*. Coexistence of myeloproliferative neoplasms with lymphoproliferative syndromes has been described in the past, whereas plasma cell dyscrasias seem to be the most common cases. *Case Presentation*. We present a case of a 59-year-old Caucasian female of Greek origin who presented with thrombocytosis. Clinical and laboratory investigation disclosed the presence of a smoldering myeloma with coexisting histological and molecular characteristics of primary myelofibrosis. The patient had the acquired point mutation V617F in the JAK2 gene but not the bcr-abl rearrangement and was treated for myelofibrosis with subsequent improvement of all haematological parameters without evidence of myelomatic evolution. *Conclusion*. We present the first case in the literature of a smoldering myeloma coexisting with primary myelofibrosis. The underlying pathogenetic mechanism could be either related to the presence of a pluripotent neoplastic stem cell capable to differentiate into both lymphoid and myeloid cells or be related to two separate nosologic entities.

## 1. Introduction

An association between plasma cell dyscrasias and myeloproliferative neoplasms has been previously described [1–5]. Coexistence of multiple myeloma (MM) and primary myelofibrosis (PMF) is considered extremely rare, whereas the association between the two clinical entities is also unclear [4, 5]. MM and CIMF are clonal proliferations of cells arising from different haemopoietic elements, and the distinction between them is based on clinical, morphological, and laboratory features [6].

In this paper, we describe a patient with clinical and pathological findings suggestive of coexisting myeloma and prefibrotic PMF.

## 2. Case Presentation

A 59-year-old Caucasian female presented with generalized malaise and headache of 7-day duration. No other symptoms were referred, and the patient was afebrile. The patient had a prior medical history of hypertension for which she was not receiving any specific treatment as well as hypothyroidism for which she was receiving levothyroxine. Physical examination was unremarkable with no organomegaly or enlarged lymph nodes. White blood cells were 10.000/*μ*L (neutrophils 7.400/*μ*L, lymphocytes 1.900/*μ*L, monocytes 300/*μ*L, and eosinophils 400/*μ*L) with normal morphology, haemoglobin was 14.0 g/dL, and platelet count was 1.042.000/*μ*L, many of which were large. Erythrocyte sedimentation rate (ESR) was 19 mm/1st hour, and clotting tests were normal. Biochemical analyses showed a raised lactate dehydrogenase (LDH = 285 IU/l) and potassium (*K* = 5.4 mEq/l). Urine analysis was unremarkable. IgG was elevated at 3300 mg/dL (normal range 701–1545 mg/dL), IgA was low at 24.5 mg/dL (normal range 48–368 mg/dL), IgM was also low at 19.3 mg/dL (normal range 25–170 mg/dL), whereas serum *β*_2_ microglobulin and C-reactive protein (CRP) were normal. There was no Bence-Jones proteinurea. Serum protein electrophoresis revealed a spike in the area of gamma globulins, and serum immunofixation showed monoclonal IgG*κ* molecule. Karyotype was normal.

There were no lytic lesions on a skeletal survey with X-rays and computed tomography (CT), whereas a skull CT showed sinusitis without any findings from the central nervous system. Gastroscopy and coloscopy did not reveal any pathology. The spleen and the liver were normal by ultrasound. Bone marrow examination revealed 15% cIgG*κ* monoclonal plasma cell infiltration [CD138(+), CD56(−)] and significant increase in the megakaryocytes population with variable appearance (markedly atypical megakaryocytes with dense clustering of naked megakaryocytic nuclei) and mild local fibrosis with little reticulin. This image could be explained in terms of a myeloproliferative neoplasm such as prefibrotic stage of myelofibrosis with concomitant existence of a plasma cell dyscrasia. Furthermore, the patient had the acquired point mutation V617F in the JAK2 gene, measured by the qualitative technique of ARMS PCR in the white blood cells of peripheral blood, but not the bcr-abl rearrangement. Since the bone marrow infiltration was only 15% with the absence of myeloma-related manifestations, the diagnosis of myelofibrosis with a smoldering IgG*κ* myeloma was made. The patient was treated with hydroxyurea 1 g/day over the first 2 months and 500 mg/day thereafter. She was also commenced on 100 mg salicylic acid daily. With this treatment, the patient's platelets have been maintained below 400.000/*μ*L and the bone marrow plasma cell infiltration at 3%, for almost five years. There is also sustained depression of the monoclonal protein concentration at levels below 2000 mg/dL.

## 3. Discussion

In the past, prefibrotic myelofibrosis could be misdiagnosed as essential thrombocythaemia (ET), and a distinction between the two entities had been greatly emphasised by histopathologists [7–9]. However, the natural history of prefibrotic myelofibrosis remains unclear, since prospective studies are lacking, and preliminary data suggest that the rate of progression to advanced disease may depend on the degree of megakaryocytic dysplasia [8, 9]. According to the latest classification of myeloproliferative neoplasms, the prefibrotic myelofibrosis without splenomegaly, no true increase of neutrophil granulopoiesis or anemia may go in favor of ET instead of PMF.

The patient presented with clinical symptoms and laboratory findings indicating the diagnosis of prefibrotic PMF. The diagnosis was documented by the trephine bone marrow biopsy which confirmed mild fibrosis along with abnormal plasma cell infiltration and absence of the Ph chromosome or the bcr-abl rearrangement [10]. Furthermore, the patient had the acquired point mutation V617F in the JAK2 gene, as has been reported by other studies [11–14]. 

Previous reports have cited an association between myelomatous bone marrow infiltration and bone marrow fibrosis and postulate that lymphokines secreted by the malignant cells may trigger the appearance of tumor-associated myelofibrosis [3–5, 15]. This concept is sustained by the known association between bone marrow fibrosis with other B-cell malignancies, such as Walderström's macroglobulinaemia and hairy cell leukaemia [1]. In contrast, other studies have suggested the coexistence of two distinct clonal diseases or the parallel, biclonal evolution of two clinical entities originating from a common stem cell [10]. Therefore, there is a distinction between patients with plasma cell dyscrasia associated with simple marrow fibrosis and patients with coexistent agnogenic myeloid metaplasia [3]. 

The patient described here has plasma cell dyscrasia documented by the existence of IgG kappa-producing plasma cells infiltrating the bone marrow and IgG paraprotein in the serum. Furthermore, the bone marrow presented hyperplasia of megakaryocyte lineage with marked atypia. This feature can be observed in MM patients as a reactive phenomenon induced by MM cells expressing factors that regulate fibroblast proliferation [16, 17]. However, the JAK2 V617F mutation excludes the probability of reactive thrombocytosis. Data suggest that the JAK2 V617F mutation does not occur in nonhematological cancers and that this mutation is uncommon in myeloid malignancies other than the classic bcr-abl-negative myeloproliferative neoplasms [18]. 

The pathogenesis underlying the coexistence of PMF and MM could be related to either the presence of a pluripotent neoplastic stem cell capable to differentiate into both lymphoid and myeloid cells or be caused by two separate neoplastic clones. The absence of myelomatic evolution after treatment for PMF may support the first hypothesis. Moreover, essential thrombocythaemia has also been reported to involve pluripotent stem cells capable to differentiate into immunoglobulin producing B-lymphoid cells and myeloid cells [19, 20]. 

We conclude that to our knowledge, this is the first case of a patient with prefibrotic PMF coexisting with a MM. Further studies are needed in order to clarify the pathophysiologic basis of this coexistence.

##  Consent 

Written informed consent was obtained from the patient for publication of this case report. A copy of the written consent is available for review by the Editor-in-Chief of this journal.

##  Conflict of Interests 

The authors declare that they have no Conflict of interests.

##  Authors' Contribution 

G. Tsirakis, P. Kanellou, M. Kaparou, and M. G. Alexandrakis were involved in the patient's care in the haematology unit, acquisition of data, analysis and interpretation of data, review of literature, and drafting the paper. A. Passam and K. Stylianou were involved in drafting and revising the paper. All authors read and approved the paper.

## List of Abbreviations

MM: Multiple myeloma
PMF: Primary myelofibrosis.
